# Timeliness gap in non‑EPI Childhood vaccination in urban China: a five-city study of socioeconomic determinants and caregiver preferences

**DOI:** 10.1186/s12939-026-02945-5

**Published:** 2026-07-10

**Authors:** Linchen Chu, Kangning Sheng, Jiajun Li, Kan Chen, Yi Zhou, Hongni Wang, Liuren Zhang, Jing Wu, Chuanxi Fu

**Affiliations:** 1 Zhejiang Chinese Medical University, No. 548, Binwen Rd, Hangzhou, 310053 China; 2 The Second School of Clinical Medicine, Zhejiang Chinese Medical University, No. 548, Binwen Rd, Hangzhou, 310053 China; 3 Center for Vaccine Impact Assessment, Key Laboratory for Quality Monitoring and Evaluation of Vaccines and Biological Products, National Medical Products Administration, No. 74, Zhongshan 2nd Rd, Guangzhou, 510080 China; 4 The Third People’s Hospital of Hubei Province, Wuhan, 430030 China; 5 Department of Vaccinology, School of Public Health, Zhejiang Chinese Medical University, Hangzhou, China

**Keywords:** Delay, Timeliness, Non‑EPI vaccines, Discrete-choice experiment, Caregiver preference, Urban China

## Abstract

**Background:**

Delays in receiving vaccinations lead to higher vaccine-preventable disease risk. We quantified the prevalence and drivers of delayed uptake of fee‑based, non-Expanded Program on Immunization (non-EPI) childhood vaccines in urban China.

**Methods:**

A multicentre clinic-based survey with an embedded discrete-choice experiment (DCE) was conducted from May to July 2024 in 15 immunisation clinics across five provincial capitals in China. Caregivers of children aged 0–6 years provided verified vaccination information, socio demographic data, scores on a 12 item Caregiver Timely Vaccination Scale, and responses to a DCEwith four attributes (price, waiting time, calendar delay, adverse event [AE] risk). Delayed vaccination was defined as receipt of at least one non-EPI dose 30 days or more after the recommended schedule.Weighted logistic regression identified correlates; mixed logit models generated marginal utilities and willingness to pay (WTP).

**Results:**

Among 1 292 valid caregiver–child pairs, the delay prevalence was 23.7% (95%CI: 21.3–26.1). Delay risk increased with monthly household income < ¥10 000 (adjusted OR: 1.24, 95% CI: 1.11–1.38), an unpleasant prior vaccination experience (1.11, 1.01–1.23) and a caregiver’s delay history (1.22, 1.10–1.36), and less likely when knowledge was adequate (0.33, 0.25–0.43). The timely scale discriminated delayed from timely caregivers (+ 1.0 mean score). In the DCE, AE risk dominated all other attributes (66.3% relative importance): lowering the stated risk from 5% to 3% increased predicted uptake by 74% points. Reducing queue time from 30 min to 10 min added 24 points. Caregivers were willing to pay ¥106 (≈ US $14) per 1% AE‑risk reduction but only ¥4 for a 10‑day improvement in calendar timeliness.

**Conclusions:**

Delayed vaccination drives non-EPI under timeliness in urban China. Embedding delay metrics and the brief 3 C screen in routine dashboards, supported by sharper AE‑risk communication and fast‑track queues, could close the gap and advance Immunization Agenda 2030 targets.

**Supplementary Information:**

The online version contains supplementary material available at https://doi.org/10.1186/s12939-026-02945-5.

## Introduction

Timely vaccination— receiving each dose within its recommended age window— remains a pre‑requisite for sustaining herd immunity and averting vaccine‑preventable diseases (VPDs). Global momentum around this metric has intensified since the World Health Organization (WHO) adopted “on‑time completion” as a keystone indicator in Immunization Agenda 2030 (IA2030) [1–3]. Yet progress has stalled: in 2023 an estimated 2.7 million children worldwide missed their third dose of diphtheria–tetanus–pertussis (DTP3) at the scheduled age—600,000 more than before the COVID‑19 pandemic [4]. Delay widens the period of susceptibility and predicts failure to finish multidose series, elevating VPD risk including measles and pertussis [5]. In contrast to vaccination coverage, assessment of vaccination timeliness can provide a more direct measure of adherence to recommendations for the ages when children should receive vaccinations.

China’s national Expanded Programme on Immunisation (EPI) maintains childhood immunization coverage above 95%, yet non‑EPI, fee‑based vaccines—such as those against rotavirus, pneumococcal disease and influenza—remain vulnerable to delays or unvaccination. One study indicated that more than 20% of children had delays in vaccinations, but no multi‑province estimate has been produced and the behavioural factors behind delay are poorly quantified. Preliminary reports implicate lower household income, conflicting information sources and fear of adverse events (AEs); however, the relative weight of each barrier is unknown [6–8]. Traditional surveys identify correlates but rarely capture how much caregivers value competing attributes when deciding whether to vaccinate on time. Discrete‑choice experiments (DCEs) bridge this gap by assigning monetary or utility values to attributes such as cost, convenience and safety. While DCEs have priced vaccine attributes in high-income settings, critical gaps remain in low- and middle-income countries (LMICs), particularly for privately funded non-routine vaccines. Most global timeliness research focuses on publicly funded routine immunization programs, with limited evidence on equity gaps in non-EPI vaccine access—where national coverage in China remains below 50% and stark socioeconomic disparities persist. No prior study has examined China’s hybrid cultural–financing context or linked stated caregiver preferences to verified vaccination records [8].

We therefore carried out a multi‑stage probability survey of caregivers at 15 immunisation clinics across five provincial capitals in mainland China (Hangzhou and Guangzhou in eastern China, Zhengzhou in central China, Chongqing and Xi’an in western China) to: (1) estimate the prevalence and socio‑demographic predictors of delayed non‑EPI vaccination among preschoolers aged 0–6 years, and (2) quantify caregivers’ willingness to pay and trade‑offs between price, convenience and safety attributes affecting on‑time uptake, using a mixed‑logit DCE.

## Methods

### Study design

A cross-sectional study with a four-stage stratified cluster sampling design, paired with a discrete choice experiment (DCE), was conducted to address the study objectives.

#### Participants

Eligible respondents were parents or other legal caregivers (e.g., grandparents) of children aged 0–6 years who presented for immunisation services during the study period (May–July 2024). For all analyses, only one index child per caregiver was included. For caregivers accompanying several children to the immunization clinic, the child receiving vaccination services during the study visit was selected as the index child, resulting in 1:1 caregiver–child pairs for the entire study sample.

### Sampling design

Given the low uptake of fee-based non-EPI vaccines across China (national coverage < 50%), which is particularly severe in rural areas, obtaining a sufficient sample size to study vaccination delays was a challenge [8]. To overcome this, we purposively focused our study on economically developed provincial capitals where higher coverage rates ensured that enough delayed doses could be observed.

#### Stage 1 (region)

Eastern, central and western macro‑regions were classified according to the *Seventh Five‑Year Plan for National Economic and Social Development*.

#### Stage 2 (city)

In each macro‑region one “national‑central” city was selected by probability‑proportional‑to‑size (PPS) using 2023 under‑seven population as weights (China Statistical Yearbook). The resulting sample comprised Guangzhou and Hangzhou (east), Zhengzhou (central) and Chongqing and Xi’an (west).

#### Stage 3 (site)

From the official immunisation‑clinic roster we randomly drew two urban community health centres and one suburban township health centre per city, giving **15 probability‑selected sites**.

#### Stage 4 (visit)

Systematic sampling (every 3rd caregiver) was used during clinic hours from May–July 2024 until city‑specific quotas (≈ 260 caregivers) were met.

A priori sample‑size calculations based on Johnson‑Orme’s rule for the DCE (*n* ≥ 750) and logistic‑regression precision indicated ≥ 1 000 caregivers were required; we enrolled 1 292.

#### Pilot survey

The questionnaire was piloted with 30 caregivers each at Puyan and Hongshan clinics (Hangzhou) to refine wording and timing.

### Instruments and quality assurance

1. Single-item arithmetic attention check.
2. Dominance test in the final DCE task; failures excluded.
3. Forms completed in < 3 min or deemed incomplete/uncooperative by interviewers were discarded.

#### 12-item caregiver timely vaccination scale

The scale was developed based on the WHO Strategic Advisory Group of Experts (SAGE) 3C Model of Vaccine Hesitancy (Complacency, Constraint, Confidence), with the addition of a Collective Responsibility dimension informed by the WHO Behavioural and Social Drivers (BeSD) of Vaccination framework and empirical research on Chinese populations vaccination decision-making. The 12‑item Timely‑Vaccination Scale yielded KMO 0.824, Bartlett *χ²*_(66)_ = 7 886.7(*P* < 0.001). Principal‑axis factoring (varimax) extracted three factors explaining 68.61% of variance: Constraint (α = 0.807), Collective‑Responsibility (α = 0.918) and Complacency (α = 0.837). A 30% hold‑out confirmatory factor analysis (CFA) showed excellent fit (CFI = 0.96; RMSEA 0.06); overall reliability α of 0.837. All items are rated on a 5-point Likert scale ranging from 0 (Strongly Disagree) to 4 (Strongly Agree). The 5 negatively worded items in the Constraint dimension are reverse-coded during data analysis to ensure consistent scoring direction across the entire scale. After reverse coding, higher total scale scores indicate more positive attitudes toward timely childhood vaccination and lower risk of delayed vaccination. The full questionnaire used for primary data collection in this study—including the 12-item scale, DCE choice tasks, and all survey items—is provided in Supplementary Table S7 of the Additional Files.

#### Outcomes

For this study, non-EPI vaccines were defined as fee-based, non-mandatory childhood vaccines not included in China’s national Expanded Programme on Immunization. These include, but are not limited to, vaccines against rotavirus infection, pneumococcal disease, influenza, varicella for age-eligible children.

#### Primary outcome

Delay = receipt of at least 1 non‑EPI dose ≥ 30 days after the Chinese CDC–recommended minimum age or scheduled interval [9].

### Study variables

#### Socio-demographic variables

**Fig. 1:**
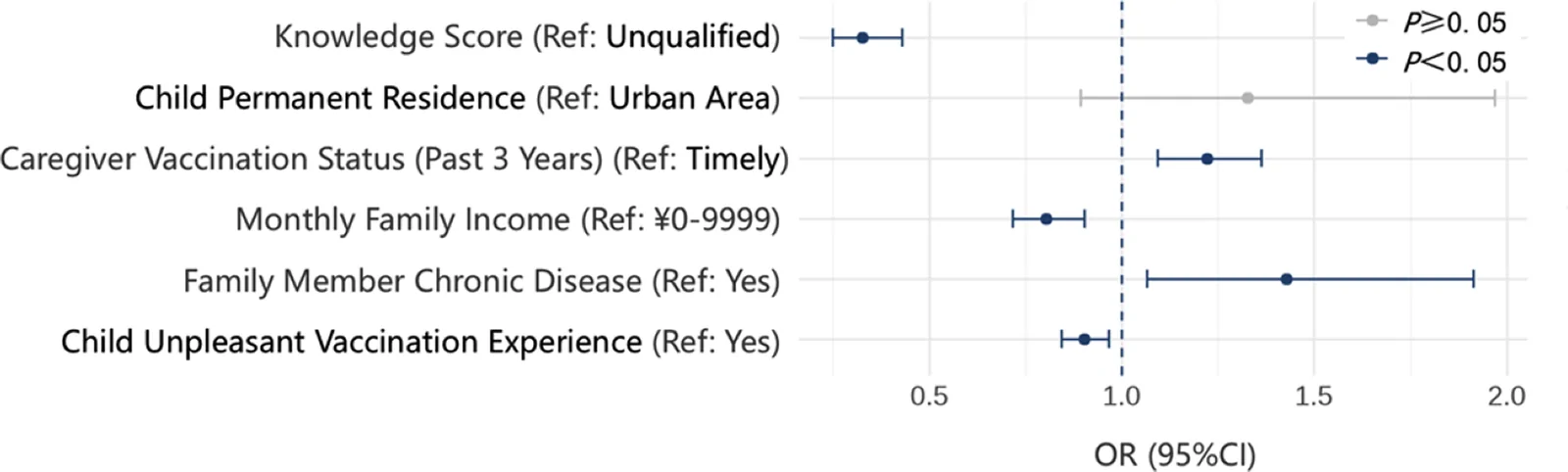
Adjusted odds ratios (aOR) for factors associated with delayed non-EPI vaccination (*n* = 1292). Note: 1. Adjustment factors included in the multivariable logistic regression model: Child-related factors: gender, birth order, permanent residence (urban/suburban), prior unpleasant vaccination experience. Caregiver-related factors: education level, occupation, past vaccination delay history (2021–2024), delayed vaccination knowledge score. Household-related factors: monthly income, number of children in the family, presence of a family member with chronic disease. 2. Reference groups for each variable are indicated in parentheses. 3. Horizontal lines represent 95% confidence intervals (CIs)

Child’s gender, birth order, permanent residence (urban/suburban), prior unpleasant vaccination experience; caregiver’s relationship to the child, education level, occupation, self-reported health status, past vaccination delay history (2021–2024); household monthly income, number of children in the family, presence of a family member with chronic disease. Reference groups for regression models are indicated in Table 1; Fig. 1.

**Table 1:** Socio-demographic characteristics of survey participants (*n* = 1292)

Variables	Category	Number	Percentage (%)
Relationship to Child	Father	323	25.0
	Mother	878	68.0
	Grandmother (Maternal/Paternal)	47	3.6
	Grandfather (Maternal/Paternal)	23	1.8
	Other	21	1.6
Caregiver Education Level	High School (Vocational School) or below	377	29.2
	College (Associate Degree)/Undergraduate or Above	915	70.8
Monthly Household Income (¥)	< 5000	280	21.7
	5000–9999	438	33.9
	10,000–19,999	328	25.4
	≥ 20,000	246	19.0
Caregiver Occupation	Agriculture/Fishing/Forestry/Livestock or Casual Lavor	97	7.5
	Healthcare Worker	158	12.2
	Government/Public Institution/Enterprise Staff (Excl. Healthcare)	215	16.6
	Retired or Unemployed	137	10.6
	Farmer	11	0.9
	Freelancer/Self-employed	345	26.7
	Enterprise/Commercial/Service Sector	329	25.5
Birth Order	First-born	843	65.2
	Second-born or above	449	34.8
Child Gender	Male	700	54.2
	Female	592	45.8
Child Residence	Urban Area	1149	88.9
	Suburban Area	143	11.1

Note. ¥ is China Yuan (1 US $=¥7.2). Birth order refers to the order of the index child’s birth; for total number of children in the family, see Table 2

#### Knowledge and attitudinal variables

Caregiver delayed vaccination knowledge score (qualified ≥ 2 points; unqualified < 2 points), scale total and subscale scores. For all monetary values, 1 US $ = ¥7.2 CNY, based on the average exchange rate during the study period (May–July 2024).

### Statistical analysis

Data were double‑entered in EpiData and analysed in SPSS 25.0 (IBM Corporation, NY, United States) and R 4.1.0 (*survey*,* mlogit mlogit*).

#### Delay prevalence

*χ²* tests for bivariate associations.

#### Determinants

Univariate and multivariable weighted logistic regression (two‑sided *P* < 0.05).

#### Preference modelling

Choice tasks (5 × 2 profiles/respondent) were analysed with mixed‑logit models estimated by maximum simulated likelihood (1 000 Halton draws). Attribute coefficients were specified as random normal except price (fixed). Model adequacy was assessed with McFadden *R²* and likelihood‑ratio tests; clustered robust SEs accounted for repeated choices. Willingness‑to‑pay (WTP) for non‑price attributes was computed as − β_attribute / β_price, where β_attribute is the estimated coefficient for the non-price attribute of interest, and β_price is the fixed coefficient for the price attribute from the mixed logit model. 95% confidence intervals for WTP values were estimated using the delta method.Relative importance of each attribute was calculated using the standard utility range method: the difference between the maximum and minimum utility values for each attribute was divided by the sum of utility ranges across all attributes. This method eliminates the bias caused by different units of measurement across attributes.

## Results

### Characteristics and delay prevalence

Of 1426 eligible caregivers, 1350 completed the questionnaire (response 94.7%). After quality‑control exclusions (*n* = 58), 1292 records were finally analysed (validity rate 95.7%). Mothers constituted 68% (weighted 65%) of respondents; 71% (68%) held ≥ undergraduate degrees. Two‑thirds of children were first‑born and weighted sex ratio was 54% boys (Table 1).

The prevalence of delay for ≥ 1 non‑EPI vaccine was 23.7% (95%CI: 21.3–26.1). Delay was more frequent among boys than girls (59.2% vs. 40.8%, *P* = 0.045) and in only‑child families than two‑child families (42.4% vs. 33.9%, *P* = 0.025). Caregiver traits associated with higher delay included education below college, farming/casual‑labour occupations and monthly income < ¥10 000 (Table 2).

**Table 2:** Basic characteristics of children by vaccination status (*n* = 1292)

Variable	Delayed Vaccination (*n* = 309, %)	Timely Vaccination (*n* = 983, %)	*P*
**Child Gender**			0.045
Male	183(59.2)	517(52.6)	
Female	126(40.8)	466(47.4)	
**Number of Children in Family**			0.025
1	161(52.1)	604(61.4)	
2	131(42.4)	333(33.9)	
3	15(4.9)	42(4.3)	
≥ 4	2(0.6)	4(0.4)	
**Caregiver Education Level**			< 0.001
Primary School or Below	29(9.4)	33(3.4)	
Junior High School / Vocational School	43(13.9)	112(11.4)	
Senior High School / Vocational College	39(12.6)	121(12.3)	
College / Undergraduate	177(57.3)	628(63.9)	
Master’s or above	21(6.8)	89(9.1)	
**Caregiver Occupation**			0.017
Agriculture/Casual Labor	38(12.3)	70(7.1)	
Healthcare Worker	40(12.9)	118(12.0)	
Government/Enterprise Staff	48(15.5)	167(17.0)	
Retired or Unemployed	39(12.6)	98(10.0)	
Freelancer/Self-employed	79(25.6)	266(27.1)	
Enterprise/Commercial/Service	65(21.0)	264(26.9)	
**Monthly Household Income (¥)**			0.019
< 5000	73(23.6)	207(21.1)	
5000–9999	89(28.8)	349(35.5)	
10,000–19,999	72(23.3)	256(26.0)	
20,000–39,999	31(10.0)	84(8.5)	
≥ 40,000	44(14.2)	87(8.9)	
**Caregiver Health Status**			0.001
Very Poor	11(3.6)	21(2.1)	
Poor	29(9.4)	40(4.1)	
Moderate	94(30.4)	281(28.6)	
Good	120(38.8)	404(41.1)	
Very Good	55(17.8)	237(24.1)	

Note. For overall socio-demographic characteristics of the entire study sample, see Table 1

### Information, reminders and satisfaction

Official hospital website were the most trusted information channel or resources, endorsed by 64% of timely vaccinators versus 59% of delayers (*P* < 0.001). Clinician advice was more influential for timely vaccinators (69% vs. 61%, *P* = 0.02). Social‑media distrust exceeded 35% in both groups.

Among delayers (*n* = 309), top reasons were the child being unwell (18%), travel (14%) and lack of caregiver time (11%); individual reasons < 10% are grouped as “other”.

Overall service satisfaction was high (94.6%), yet lower in the delayed group (91.3%) than the timely group (95.6%; absolute difference 4.3 pp; OR = 1.7, 95%CI: 1.1–2.5). Unpleasant past vaccination experiences were reported by half of delayers compared with one-third of timely vaccinators (*P* < 0.001). See Additional file 1 (Table S1).

### Caregiver attitudes

Exploratory factor analysis (KMO 0.824) yielded three components—*Constraint*,* Collective responsibility*,* Complacency*—explaining 69% of variance; confirmatory factor analysis showed good fit (CFI 0.96; RMSEA 0.06). Timely vaccinators scored higher on the total 3C scale (mean difference 1.0 points, 95% CI 0.4–1.7) and on *Constraint* and *Collective-responsibility* subscales (Table 3 and Additional file 1, Tables S2-S3).

**Table 3:** Caregiver timely vaccination scale scores by vaccination status (*n* = 1292)

Scale	Delayed Vaccination (*n* = 309)	Timely Vaccination (983)	*P*
Total Scale Score	42.87 (5.81)	43.90 (6.11)	0.002
Constraint	15.20 (4.04)	15.67 (4.02)	0.039
Collective Responsibility	12.09 (2.41)	12.50 (2.28)	0.002
Complacency	15.57 (3.13)	15.73 (3.21)	0.366

Note: Scores expressed as Mean (Standard Deviation)

### Multivariable determinants of delay

Figure 1 presents adjusted odds ratios (aOR). After including the 6 factors with statistical significance from the univariate analysis into the multivariable model, the results showed that children with unpleasant prior vaccination experience, no family members with chronic diseases, lower monthly household income (<¥10 000), caregivers with a history of vaccination delay in past three years (2021–2024), and caregivers with unqualified delayed vaccination knowledge scores were more likely to experience delayed vaccination.

Compared with children living in urban areas, children living in suburban areas seemed to have delayed vaccination risk of aOR = 1.327 (95%CI: 0.894–1.968, *P* = 0.160). Compared with children with unpleasant prior vaccination experience, children without such experience had significantly lower risk of delayed vaccination (aOR = 0.903, 95%CI: 0.844–0.966, *P* = 0.003). Compared with children who had family members with chronic diseases, children without family members with chronic diseases had significantly increased risk of delayed vaccination (aOR = 1.427, 95%CI: 1.065–1.913, *P* = 0.017). Compared with children from households with monthly income <¥10 000, children from households with monthly income ≥¥10 000 had significantly lower risk of delayed vaccination (aOR = 0.804, 95%CI: 0.717–0.902, *P* < 0.001). Children whose caregivers had a history of vaccination delay in the past three years had a significantly increased risk of delayed vaccination (aOR = 1.221, 95%CI: 1.094–1.362, *P* < 0.001). Children whose caregivers had qualified delayed vaccination knowledge scores had a significantly lower risk of delayed vaccination (aOR = 0.327, 95%CI: 0.249–0.429, *P* < 0.001). See also Additional file 1 (Table S5).

### Discrete-choice experiment: attribute importance and WTP

**Fig. 2:**
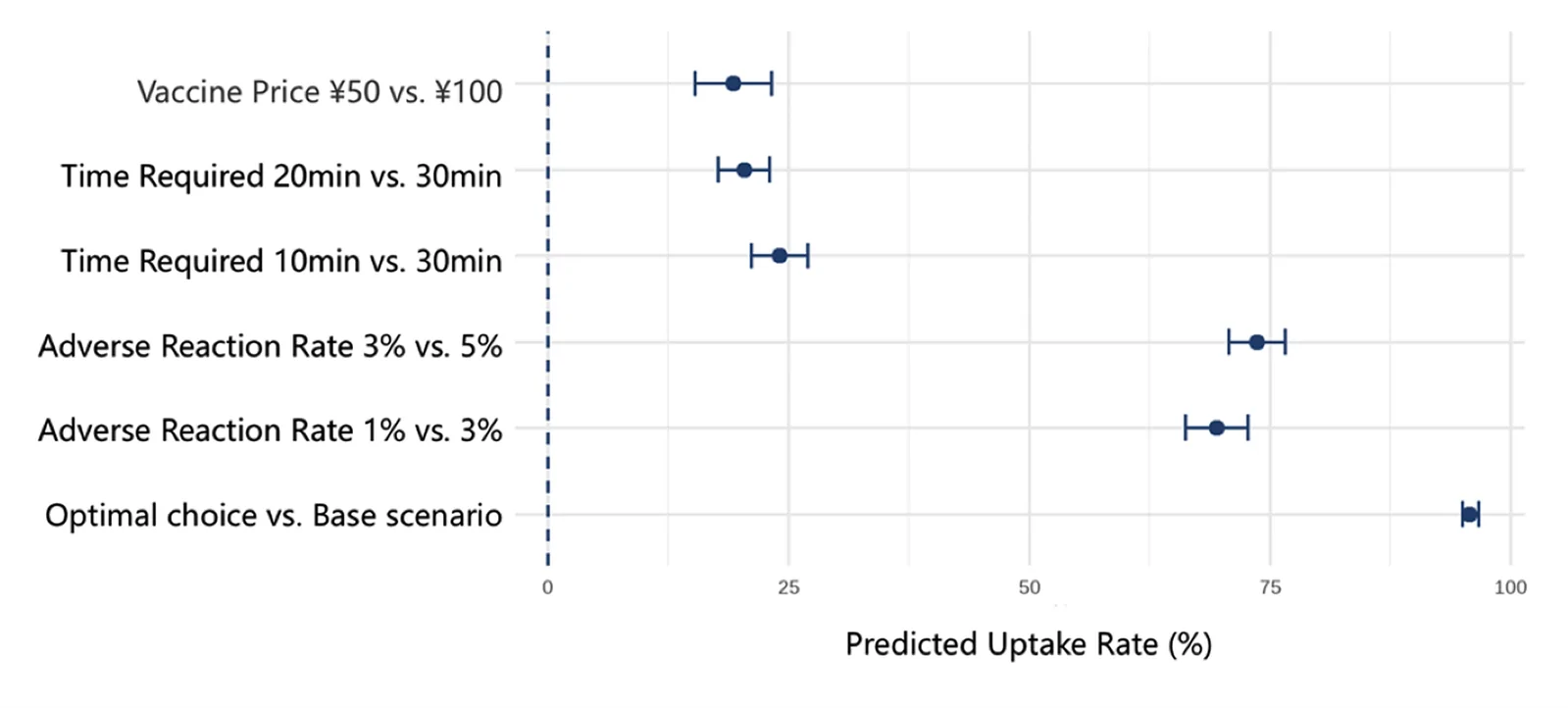
Effect of attribute level change on expected vaccination rates

Mixed-logit analysis fit the data well (–2 Log‑likelihood = 5479; McFadden R² 0.28; *P* < 0.001) and 72% of choices correctly predicted. Adverse-event rate dominated preferences (relative importance 66.3%), followed by price (15.8%), waiting time (14.7%) and calendar days (3.2%) (Fig. 2 and Additional file 1, Table S6).

Changing a single attribute from the base scenario (60 day delay, 30 min wait, ¥100 price, 5% AE) produced: AE rate: lowering to 3% uptake by + 74 pp; to 1% a further + 70 pp. Waiting time: cutting from 30 min to 10 min uptake by + 24 pp. Price: halving to ¥50 uptake by + 19 pp. Calendar delay: eliminating the 60‑day lag had negligible effect (+ 0.1 pp). Combining best levels (1% AE, 10‑min wait, free vaccine, no delay) yielded a projected uptake of 96% (95%CI: 95–97).

Caregivers were willing to pay ¥106 (≈ US $14; 95%CI: 98–114) for each 1% reduction in AE risk and ¥47 (41–53) for every 10‑minute decrease in clinic time, but only ¥4 (1–6) for each 10‑day reduction in calendar delay. Full coefficients and scenario analyses appear in Additional file 1 (Table S6).

## Discussion

This five‑capital-city survey of caregivers in 15 clinics found that nearly one in four urban preschoolers was behind schedule for at least one fee‑based, non‑EPI vaccine. Scaled to China’s 42 million 0–6‑year‑olds who live in provincial‑level cities, the burden exceeds 10 million children and roughly 45 million delayed doses each year. These numbers expose a sizeable blind spot in China’s otherwise strong immunisation profile and underline the public‑health value of measuring when vaccines are given, not merely whether they are ultimately received.

Socio-economic fault-lines drive much of this gap, consistent with a broad international literature documenting income-related and psychological barriers to equitable immunization access in both high-income and low-resource settings [10–12]. Our study provides novel evidence from the world’s largest national immunization system, demonstrating that monthly income below ¥10,000, an unpleasant prior vaccination experience, and a caregiver’s personal history of delay each raised risk by 11–24%, whereas a qualified knowledge score cut it by two-thirds. Our DCE finding that adverse event risk perceptions dominate decision-making contributes generalizable insights to global behavioral economics research on vaccine hesitancy, a key barrier to achieving the WHO Immunization Agenda 2030 target of 90% on-time childhood vaccination completion. These structural and cognitive levers outweighed simple birth‑order or urban‑suburban residence effects, highlighting the need to look beyond demographic averages [10, 11, 13].

A streamlined 12‑item attitude scale confirmed that constraints on time and a sense of collective responsibility—not complacency—distinguish timely from delayed caregivers. Although the effect size was modest (mean difference + 1.0 point), the scale’s brevity makes it a practical triage tool for identifying families who would benefit most from fast‑track services or focused counselling.

The information ecosystem amplifies these disparities. Timely vaccinators placed greater trust in hospital/CDC websites and clinician advice, whereas delayers navigated noisier social‑media streams that one‑third openly distrusted [14]. Strengthening provider‑led digital communication therefore remains crucial for neutralising misinformation at scale [14–20].

Behavioural‑economic evidence points to two high‑yield service levers. First, perceived serious adverse‑event (AE) risk overwhelmingly shapes decisions, accounting for 66.3% of total attribute weight in our discrete‑choice experiment. Lowering the stated AE rate from 5% to 3% raised projected uptake by 74% points—triple the gain achieved by cutting clinic wait‑time from 30 to 10 min. Second, Consistent with the relative importance results, caregivers were willing to pay ¥106 (≈ US $14; 95%CI: 98–114) for each 1% reduction in AE risk, ¥47 (95% CI 41–53) for every 10-minute decrease in clinic waiting time, and only ¥4 (95%CI: 1–6) for each 10-day reduction in calendar delay. These WTP values were calculated directly from the mixed logit model coefficients presented in Supplementary Table S6 (price coefficient: β=−0.009, *P* < 0.001; AE risk coefficient: β=−0.944, *P* < 0.001; waiting time coefficient: β=−0.042, *P* < 0.001; calendar delay coefficient: β=−0.003, *P* = 0.090), using the standard WTP formula detailed in the Methods section. These asymmetries suggest that vivid, data‑driven risk communication and strict pharmacovigilance transparency will produce outsized returns compared with reminder messages alone [21, 22].

Besides the coverage traditionally, delay should be considered to be surveillance nationally. Timeliness indicators would (i) reveal neighbourhood‑level susceptibility windows, (ii) trigger SMS nudges precisely when protection lapses, and (iii) spotlight equity gaps—low‑income and multi‑child households in our data were nearly twice as likely to fall behind. Incorporating “percent of non‑EPI doses given on time” into national dashboards, and embedding the timely vaccination screen in electronic health‑records, would convert these insights into routine programme action.

Policy priorities emerge clearly. Income‑linked vouchers can offset price sensitivity; the tangible constraint of time can be addressed through appointment apps and 10-minute wait-time targets; and standardised AE‑risk infographics, paired with mandatory counselling prompts, can recalibrate caregiver perceptions. Together, such interventions address both the economic and psychological barriers highlighted by our models [15, 23, 24].

While we focus on urban areas—where the absolute burden of non-EPI vaccine use are high—rural children face even more severe structural barriers to non-EPI vaccination access and timeliness in China. These include lower household income, fewer immunization clinics per capita, geographic barriers to care, reduced access to official vaccine information, and lower health literacy, all of which exacerbate rural-urban immunization inequities consistent with national and international evidence. Future research priorities should include: (1) nationally representative studies including rural, remote, and underserved groups to estimate national non-EPI vaccine delay prevalence and quantify urban-rural equity gaps; (2) urban-rural comparative analyses of timeliness determinants to identify context-specific barriers; (3) mixed-methods studies investigating structural barriers to rural vaccination access; and (4) intervention studies testing targeted, equity-focused strategies to improve non-EPI vaccine timeliness in rural populations, aligned with the IA2030 goal of leaving no one behind.

There are limitations in the study. Cross-sectional data permit association, not causation; however, income and prior experience plausibly precede vaccination decisions. Urban sampling limits rural inference, though urban delay carries the larger absolute burden of optional doses, the expanded discussion above outlines critical future studies on rural population. Recall bias is possible. Finally, stated preferences may over-predict behaviour, but dominance checks and realistic attribute levels reduce hypothetical bias.

In sum, delay—rather than refusal—is the challenge for non‑EPI vaccines in China’s urban centres. Economic pressure, risk misperception and service bottlenecks explain far more of the gap than caregiver complacency. Embedding delay surveillance into routine systems, while coupling risk‑communication upgrades with queue‑management and targeted subsidies, will offer a feasible route to closing the timeliness gap and advancing China’s contribution to the Immunization Agenda 2030 goal of 90% on‑time completion.

## Supplementary Information

Below is the link to the electronic supplementary material.

Supplementary Material 1: Additional file 1: Supplementary tables and questionnaire. This file contains seven supplementary tables, including comparisons between timely and delayed vaccination groups (Table S1), exploratory factor analysis results (Table S2), caregiver 3C scale scores by baseline characteristics (Table S3), univariate and multivariate logistic regression analyses of child vaccination status (Tables S4-S5), mixed logit model results for non-EPI vaccination scenario preferences (Table S6), and the full questionnaire on child vaccination delay behavior, attitudes, and decision-making (Table S7), which was the primary data collection instrument for this study.

## Data Availability

The datasets generated and analysed during the current study are not publicly available due to concerns regarding participant privacy and ethical restrictions associated with the informed consent process, but are available from the corresponding author on reasonable request.

## References

[1] Clark A, Sanderson C. Timing of children’s vaccinations in 45 low-income and middle-income countries: an analysis of survey data[J]. Lancet. 2009;373(9674):1543–9. 10.1016/S0140-6736(09)60317-2 19303633

[2] Rane MS, Rohani P, Halloran ME. Association of Diphtheria-Tetanus–Acellular Pertussis Vaccine Timeliness and Number of Doses With Age-Specific Pertussis Risk in Infants and Young Children[J]. JAMA Netw Open. 2021;4(8):e2119118. 10.1001/jamanetworkopen.2021.19118 PMC8356064 34374773

[3] Lindstrand A, Mast E, Churchill S, et al. Implementing the immunization agenda 2030: A framework for action through coordinated planning, monitoring & evaluation, ownership & accountability, and communications & advocacy. Vaccine. 2024;42(Suppl 1Suppl 1):S15–27. 10.1016/j.vaccine.2021.09.045 PMC10801759 36639274

[4] Message by the Director of the Department of Immunization, Vaccines and Biologicals at WHO - June 2024[EB/OL]. 2025 Feb 8. https://www.who.int/news/item/16-07-2024-message-by-the-director-of-the-department-of-immunization--vaccines-and-biologicals-at-who---june-2024.

[5] Akmatov MK, Mikolajczyk RT. Timeliness of childhood vaccinations in 31 low and middle-income countries[J]. J Epidemiol Commun H. 2012;66(7):e14. 10.1136/jech.2010.124651 21551179

[6] Campbell H, Edwards A, Letley L, et al. Changing attitudes to childhood immunisation in English parents[J]. Vaccine. 2017;35(22):2979–85. 10.1016/j.vaccine.2017.03.089 28442229

[7] Wang X, Fan Y, Wang W. Investigation of non-National Immunization Program vaccination intentions in rural areas of China[J]. BMC Public Health. 2023;23:1485. 10.1186/s12889-023-16390-4 PMC10401748 37542236

[8] Zhang H, Lai X, Mak J, et al. Coverage and Equity of Childhood Vaccines in China. JAMA Netw Open. 2022;5(12):e2246005. Published 2022 Dec 1. 10.1001/jamanetworkopen.2022.46005 PMC9856225 36484985

[9] Wang Q, Xiu S, Yang L, et al. Delays in routine childhood vaccinations and their relationship with parental vaccine hesitancy: a cross-sectional study in Wuxi, China. Expert Rev Vaccines. 2022;21(1):135–43. 10.1080/14760584.2022.2008244 34789062

[10] Lucane Z, Kursite M, Sablinskis K, et al. COVID-19 Vaccination Coverage and Factors Influencing Vaccine Hesitancy among Patients with Inborn Errors of Immunity in Latvia: A Mixed-Methods Study[J]. Vaccines. 2023;11(11):1637. 10.3390/vaccines11111637 PMC10675738 38005969

[11] Brewer NT, Chapman GB, Rothman AJ, et al. Increasing Vaccination: Putting Psychological Science Into Action[J]. Psychol Sci Public Interest. 2017;18(3):149–207. 10.1177/1529100618760521 29611455

[12] Freeman D, Lambe S, Yu L-M, et al. Injection fears and COVID-19 vaccine hesitancy[J]. Psychol Med. 2023;53(4):1185–95. 10.1017/S0033291721002609 PMC8220023 34112276

[13] Olusanya OA, Bednarczyk RA, Davis RL, et al. Addressing parental vaccine hesitancy and other barriers to childhood/adolescent vaccination uptake during the coronavirus (COVID-19) pandemic[J]. Front Immunol. 2021;12:663074. 10.3389/fimmu.2021.663074 PMC8012526 33815424

[14] Abdullahi LH, Kagina BM, Ndze VN, et al. Improving vaccination uptake among adolescents[J]. Cochrane Database Syst Rev. 2020;2020(1):CD011895. 10.1002/14651858.CD011895.pub2 PMC6984618 31978259

[15] Chen H, Li X, Gao J, et al. Health Belief Model Perspective on the Control of COVID-19 Vaccine Hesitancy and the Promotion of Vaccination in China: Web-Based Cross-sectional Study[J]. J Med Internet Res. 2021;23(9):e29329. 10.2196/29329 PMC8425399 34280115

[16] Cataldi JR, O’Leary ST. Parental vaccine hesitancy: scope, causes, and potential responses[J]. Curr Opin Infect Dis. 2021;34(5):519–26. 10.1097/QCO.0000000000000774 34524202

[17] Hu Y, Liang H, Wang Y, et al. Inequities in childhood vaccination coverage in Zhejiang, Province: evidence from a decomposition analysis on two-round surveys[J]. Int J Env Res Pub He. 2018;15(9):2000. 10.3390/ijerph15092000 PMC6164817 30217080

[18] Larson HJ, Emmanuela Gakidou CJL, Murray. Vaccine-Hesitant Moment[J]. 2022;387(1):58–65. 10.1056/NEJMra2106441 PMC9258752 35767527

[19] van der Linden S. We need a gold standard for randomised control trials studying misinformation and vaccine hesitancy on social media[J]. BMJ. 2023;381:1007. 10.1136/bmj.p1007 37146997

[20] Zhang L, Chu L, Sundaram ME, et al. Identifying Preferred Features of Influenza Vaccination Programs Among Chinese Clinicians Practicing Traditional Chinese Medicine and Western Medicine: Discrete Choice Experiment[J]. JMIR Public Health Surveill. 2025;11:e63314–63314. 10.2196/63314 PMC11769775 39864957

[21] Hou Z, Tong Y, Du F, et al. Assessing COVID-19 Vaccine Hesitancy, Confidence, and Public Engagement: A Global Social Listening Study[J]. J Med Internet Res. 2021;23(6):e27632. 10.2196/27632 PMC8202656 34061757

[22] Wong EL, Qiu H, Chien WT, et al. COVID-19 Vaccine Willingness and Related Factors Among Health Care Workers in 3 Southeast Asian Jurisdictions[J]. JAMA Netw Open. 2022;5(8):e2228061. 10.1001/jamanetworkopen.2022.28061 PMC9396363 35994284

[23] Leal P, Gaete J, González C, et al. Delays in the vaccination of infants between 2 and 18 months of age: associated factors in Chile[J]. BMC Public Health. 2023;23(1):1882. 10.1186/s12889-023-16769-3 PMC10540413 37770902

[24] Peters MDJ. Addressing vaccine hesitancy and resistance for COVID-19 vaccines[J]. Int J Nurs Stud. 2022;131:104241. 10.1016/j.ijnurstu.2022.104241 PMC8972969 35489108

