# Supplementary material for: Timeliness gap in non‑EPI Childhood vaccination in urban China: a five-city study of socioeconomic determinants and caregiver preferences
Source: Int J Equity Health. 2026 Jul 10;25:234. doi: 10.1186/s12939-026-02945-5 (PMC13640121; doi:10.1186/s12939-026-02945-5)
Supplement: Supplementary file 1 [file 12939_2026_2945_MOESM1_ESM.docx]

**Appendix**

Supplementary Table S1 Comparison of Experiences between Timely and Delayed Vaccination Groups (n=1292)

| **Variable** | **Delayed Vaccination (n=309, %)** | **Timely Vaccination (n=983, %)** | **P** |
| --- | --- | --- | --- |
| **Most trusted information channel** |  |  | <0.001 |
| Hospital Official Website | 182 (58.9) | 624 (63.5) |  |
| Traditional Media | 14 (4.5) | 33 (3.4) |  |
| Social Media Platforms | 15 (4.9) | 17 (1.7) |  |
| Parenting Apps/Mom Groups | 12 (3.9) | 17 (1.7) |  |
| Search Engines | 8 (2.6) | 12 (1.2) |  |
| Healthcare Workers | 68 (22.0) | 253 (25.7) |  |
| Family/Friends | 10 (3.2) | 27 (2.7) |  |
| **Most distrusted information channel** |  |  | <0.001 |
| Hospital Official Website | 52 (16.8) | 91 (9.3) |  |
| Traditional Media | 19 (6.1) | 53 (5.4) |  |
| Social Media Platforms | 109 (35.3) | 368 (37.4) |  |
| Parenting Apps/Mom Groups | 33 (10.7) | 104 (10.6) |  |
| Search Engines | 74 (23.9) | 266 (27.1) |  |
| Healthcare Workers | 6 (1.9) | 20 (2.0) |  |
| Family/Friends | 16 (5.2) | 81 (8.2) |  |
| **Satisfaction with Vaccination Service** |  |  | 0.01 |
| Dissatisfied | 8 (2.6) | 16 (1.6) |  |
| Moderate | 19 (6.1) | 27 (2.7) |  |
| Satisfied | 282 (91.3) | 940 (95.6) |  |
| **Unpleasant Vaccination Experience** |  |  | <0.001 |
| Yes | 156 (50.5) | 344 (35.0) |  |
| No | 153 (49.5) | 639 (65.0) |  |
| **Delay Experience in Past 3 Years** |  |  | <0.001 |
| 0 times | 88 (28.5) | 443 (45.1) |  |
| 1-2 times | 150 (48.5) | 374 (38.0) |  |
| ≥3 times | 15 (4.9) | 35 (3.6) |  |
| Uncertain | 35 (11.3) | 82 (8.3) |  |
| Not Vaccinated | 21 (6.8) | 49 (5.0) |  |
| **Delayed Vaccination Knowledge Score** |  |  | 0.039 |
| Unqualified | 96 (31.1) | 247 (25.1) |  |
| Qualified | 213 (68.9) | 736 (74.9) |  |

Supplementary Table S2 Exploratory Factor Analysis of Caregiver Timely Vaccination Scale (n=1292)

| **Dimension** | **Item** | **Std. Factor Loading** | **Cronbach's α** |
| --- | --- | --- | --- |
| **Constraint** | Taking my child for vaccination takes up a lot of my time | 0.799 | 0.807 |
|  | Unreasonable vaccination clinic hours affect my ability to take my child for vaccination. | 0.796 |  |
|  | Suggestions from family members or friends cause me to delay my child's vaccination. | 0.792 |  |
|  | The process of coming to get vaccinated increases the risk of my child getting infected by others. | 0.747 |  |
|  | For a new vaccine, I tend to postpone the vaccination time. | 0.609 |  |
| **Collective Responsibility** | I should listen to the doctor and vaccinate my child on time. | 0.902 | 0.918 |
|  | I will actively respond to national policies and vaccinate my child on time. | 0.870 |  |
|  | To protect my family, I will take my child for timely vaccination. | 0.858 |  |
| **Complacency** | Delayed vaccination reduces my child's immunity to infectious diseases. | 0.812 | 0.837 |
|  | Timely vaccination for my child is important for the health of people around us. | 0.773 |  |
|  | Timely vaccination is important for my child's health. | 0.766 |  |
|  | My child being sick affects my ability to take them for timely vaccination. | 0.763 |  |

*Note: Total scale Cronbach's α = 0.837; KMO = 0.824; Bartlett's Test significant (*P*<0.001).*

Supplementary Table S3 Caregiver '3C' Scale Scores by Baseline Characteristics (n=1292)

|  | | **Total Score Mean (SD)** | **The average score of each dimension** | | |
| --- | --- | --- | --- | --- | --- |
|  |  |  | **Constraint** | **Collective Responsibility** | **Complacency** |
| **Gender** | Male | 43.37 (0.22) | 15.45 (0.15) | 12.27 (0.09) | 15.65 (0.12) |
|  | Female | 43.28 (0.25) | 15.35 (0.17) | 12.29 (0.09) | 15.64 (0.12) |
| **Birth Order** | First-born | 43.19 (0.21) | 15.30 (0.14) | 12.33 (0.08) | 15.56 (0.11) |
|  | Second-born | 43.57 (0.29) | 15.61 (0.20) | 12.15 (0.12) | 15.81 (0.14) |
|  | Third-born | 43.83 (0.81) | 15.71 (0.60) | 12.43 (0.39) | 15.69 (0.46) |
| **Residence** | Urban | 43.57 (0.32) | 15.57 (0.22) | 12.24 (0.12) | 15.76 (0.17) |
|  | Rural | 43.25 (0.19) | 15.35 (0.13) | 12.29 (0.08) | 15.60 (0.10) |
| **Family Chronic Disease Member** | Yes | 43.18 (0.30) | 15.27 (0.21) | 12.20 (0.12) | 15.71 (0.16) |
|  | No | 43.40 (0.20) | 15.48 (0.13) | 12.31 (0.08) | 15.62 (0.11) |
| **Caregiver Education** | High School or Below | 43.40 (0.30) | 15.45 (0.20) | 12.25 (0.12) | 15.70 (0.16) |
|  | College/Undergraduate | 43.39 (0.21) | 15.40 (0.15) | 12.34 (0.08) | 15.65 (0.12) |
|  | Master's or Above | 42.65 (0.53) | 15.32 (0.37) | 11.91 (0.23) | 15.43 (0.27) |
| **Monthly Family Income (¥)** | 0-9999 | 43.31 (0.21) | 15.27 (0.15) | 12.30 (0.09) | 15.74 (0.11) |
|  | 10000-39999 | 43.33 (0.30) | 15.58 (0.20) | 12.23 (0.11) | 15.51 (0.16) |
|  | ≥40000 | 43.40 (0.50) | 15.56 (0.41) | 12.27 (0.22) | 15.57 (0.31) |

*Note: Scores expressed as Mean (Standard Deviation).*

Supplementary Table S4 Univariate Logistic Regression Analysis of Child Vaccination Status (n=1292)

| **Influencing Factor** | **Odds Ratio** | **95% CI** | **P** |
| --- | --- | --- | --- |
| **Child Permanent Residence** |  |  |  |
| Urban Area | 1.000 |  |  |
| Suburban Area | 1.822 | 1.637 - 2.007 | 0.001 |
| **Child Unpleasant Vaccination Experience** |  |  |  |
| Yes | 1.000 |  |  |
| No | 0.920 | 0.887 - 0.953 | 0.011 |
| **Family Member Chronic Disease** |  |  |  |
| Yes | 1.000 |  |  |
| No | 1.472 | 1.331 - 1.613 | 0.006 |
| **Monthly Family Income (¥)** |  |  |  |
| 0-9999 | 1.000 |  |  |
| ≥10000 | 0.791 | 0.736 - 0.846 | <0.001 |
| **Caregiver Vaccination Status (Past 3 Years)** |  |  |  |
| Timely | 1.000 |  |  |
| Delayed | 1.231 | 1.178 - 1.284 | <0.001 |
| **Knowledge Score** |  |  |  |
| Unqualified | 1.000 |  |  |
| Qualified | 0.296 | 0.160 - 0.432 | <0.001 |

Supplementary Table S5 Multivariate Logistic Regression Analysis of Child Vaccination Status (n=1292)

|  | **Odds Ratio** | **95% CI** | P |
| --- | --- | --- | --- |
| **Child Permanent Residence** |  |  |  |
| Urban Area | 1.000 |  |  |
| Suburban Area | 1.327 | 0.894 - 1.968 | 0.160 |
| **Child Unpleasant Vaccination Experience** |  |  |  |
| Yes | 1.000 |  |  |
| No | 0.903 | 0.844 - 0.966 | 0.003 |
| **Family Member Chronic Disease** |  |  |  |
| Yes | 1.000 |  |  |
| No | 1.427 | 1.065 - 1.913 | 0.017 |
| **Monthly Family Income (¥)** |  |  |  |
| 0-9999 |  |  |  |
| ≥10000 | 0.804 | 0.717 - 0.902 | <0.001 |
| **Caregiver Vaccination Status (Past 3 Years)** |  |  |  |
| Timely | 1.000 |  |  |
| Delayed | 1.221 | 1.094 - 1.362 | <0.001 |
| **Knowledge Score** |  |  |  |
| Unqualified | 1.000 |  |  |
| Qualified | 0.327 | 0.249 - 0.429 | <0.001 |

Supplementary Table S6 Mixed Logit Main Effects Model Regression Analysis for Child Non-EPI Vaccination Scenario Preferences

| **Attribute** | **β (95% CI)** | **Relative Importance (%)** | **Std. Error** | **z** | **P** |
| --- | --- | --- | --- | --- | --- |
| **Vaccine Price (¥)** | -0.009 (-0.011, -0.007) | 15.8 | 0.0009 | -9.61 | <0.001 |
| **Waiting Time (min)** | -0.042 (-0.047, -0.036) | 14.7 | 0.0028 | -14.68 | <0.001 |
| **calendar delay** | -0.003 (-0.007, 0.001) | 3.2 | 0.0019 | -1.70 | 0.090 |
| **Adverse Reaction Rate (%)** | -0.944 (-1.008, -0.880) | 66.3 | 0.0326 | -28.93 | <0.001 |

Supplementary Table S7 Questionnaire on Child Vaccination Delay Behavior, Attitudes, and Decision-Making

The questionnaire comprised four sections (44 questions total):

1. Basic Information: Child (gender, DOB, birth order, health, vaccination status) and caregiver (education, occupation, income, health). Child vaccination status ("Timely"/"Delayed") was based on selecting "catch-up vaccination" (indicating missed/delayed dose).

2. Delayed Vaccination Experiences & Knowledge: Information sources/trust, reasons for child/caregiver delay/catch-up, post-delay emotions (worry, anger; 0-10 scale, >5 negative), post-catch-up emotions (satisfaction, etc.), service satisfaction, past experiences, delay frequency, knowledge (7 MCQs, score: +1 correct, -1 incorrect, 0 unsure/uncertain; qualified ≥2 correct).

3. Caregiver Timely Vaccination Scale (3C): Adapted from original 3C scale using Delphi method and pilot testing, adding "Collective Responsibility." 12 items: Constraint (5), Collective Responsibility (3), Complacency (4). Constraint: Practical barriers (social factors, time, risk concerns). Collective Responsibility: Consideration of societal/family welfare in decision-making. Complacency: Underestimating necessity/importance (low perceived risk). 5-point Likert scale (0=Strongly Disagree to 4=Strongly Agree). Constraint items reverse-scored. Higher subscale/total scores indicate stronger agreement with the construct/positive timely vaccination attitude.

4. Scenario Choice (DCE): Preferences for non-NIP vaccination (Influenza vaccine example). 5 choice sets, each with 2 options. Attributes/levels (Table 1) determined by literature, qualitative interviews, Delphi method. Orthogonal design used.

Attributes and Levels for Vaccination Choice Preference DCE

| **Vaccine Attribute** | **Levels** |
| --- | --- |
| **Delay Required (days)** | 0 / 30 / 60 |
| **Time to Vaccination Site (min)** | 10 / 20 / 30 |
| **Vaccination Cost (¥)** | 0 / 50 / 100 |
| **Adverse Event[AE] Risk (%)** | 1 / 3 / 5 |
